# ESR Innovation in Focus: From technological innovations to clinical impact

**DOI:** 10.1007/s00330-026-12362-5

**Published:** 2026-02-13

**Authors:** Konstantin Nikolaou

**Affiliations:** Department of Diagnostic and Interventional Radiology, University Hospital Tübingen, Tübingen, Germany

Radiology stands at the center of a rapidly transforming medical landscape, driven by unprecedented technological progress. Innovations in imaging, data science, and digital infrastructures are reshaping how we diagnose disease, guide therapy, conduct research, and educate the next generation of radiologists. In this dynamic environment, radiologists must not only adopt new technologies but also actively shape their responsible and meaningful integration into clinical practice.

Constant innovation is an integral part of everyday radiology. Artificial intelligence, advanced imaging techniques, novel interventional approaches, and interoperable digital ecosystems are already directly influencing our workflows, decision-making, and patient outcomes. Navigating this complexity requires critical appraisal, strategic vision, and a clear understanding of how innovation can add real value to patient care.

In 2026, *European Radiology* is launching a new publication series entitled “ESR Innovation in Focus”. This series is dedicated to advancing knowledge on the latest radiological and technical innovations and to providing strategic direction on their implementation in clinical practice, research, and education. “ESR Innovation in Focus” will highlight key developments within a rapidly evolving technological landscape, where radiology continues to be at the forefront of transformative change.

Through expert perspectives and focused contributions, this series will help readers identify meaningful innovations, understand their clinical relevance, and anticipate future directions. By doing so, “ESR Innovation in Focus” aims to support radiologists in responsibly leading innovation and ensuring that technological progress translates into improved care, robust research, and high-quality education for the benefit of our patients and the radiological community.

